# Communicating carabids: Engaging farmers to encourage uptake of integrated pest management

**DOI:** 10.1002/ps.6878

**Published:** 2022-04-07

**Authors:** Kelly Jowett, Alice E Milne, Simon G Potts, Deepa Senapathi, Jonathan Storkey

**Affiliations:** ^1^ Rothamsted Research Hertfordshire UK; ^2^ Sustainable Agricultural Sciences Rothamsted Research Hertfordshire UK; ^3^ Centre for Agri‐Environmental Research School of Agriculture Policy and Development, University of Reading Reading UK

**Keywords:** agri‐environment schemes, carabid beetles, theory of planned behaviour, conservation biocontrol, questionnaire, engagement

## Abstract

**BACKGROUND:**

Natural enemy pest control is becoming more desirable as restrictions increase on pesticide use. Carabid beetles are proven agents of natural‐enemy pest control (NPC), controlling pests and weeds in crop areas. Agro‐ecological measures can be effective for boosting carabid abundance and associated NPC, but the benefits of specific interventions to production are seldom communicated to farmers. We explore pathways to improved NPC by engaging farmers and increasing knowledge about farm management practices (FMPs) beneficial to carabids using engagement materials. We used a questionnaire to measure awareness, beliefs and attitudes to carabids and analysed these within a framework of the Theory of Planned Behaviour (TPB), relative to a control group.

**RESULTS:**

We found awareness of carabid predation to be associated with beliefs of pest and weed control efficacy. Within the framework of TPB, we found that current implementation of FMPs was higher if farmers perceived them to be both important for carabids and easy to implement. This was also true for future intention to implement, yet the perceived importance was influenced by engagement materials. Field margins/buffer strips and beetle banks (16% and 13% of responses) were the most favoured by farmers as interventions for carabids.

**CONCLUSION:**

The TPB is a valuable tool with which to examine internal elements of farmer behaviour. In this study self‐selected participants were influenced by online engagement in a single intervention, proving this approach has the potential to change behaviour. Our results are evidence for the effectiveness of raising awareness of NPC to change attitudes and increase uptake of sustainable practices.

## Graphical abstract

Engagement interventions affected behavioural intentions of farmer participants primarily through altering beliefs about carabid pest control, resulting in more positive attitudes towards specific farm management practices.

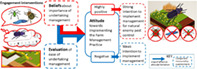

© 2022 The Authors. *Pest Management Science* published by John Wiley & Sons Ltd on behalf of Society of Chemical Industry.

## 1 INTRODUCTION

Agri‐environment schemes (AESs) were introduced in the UK to mitigate the negative environmental impacts of the expansion and industrialisation of agriculture.^1^ These schemes provide funding to farmers and land managers to farm in a way that supports biodiversity, enhances the landscape and improves the quality of water, air and soil. However, since these schemes are voluntary and payments are currently not based on quality of implementation, outcomes have been variable.^2^ It has been shown that when practitioners understand the premise and appreciate the benefits of a course of action, they are more likely to implement it effectively.^3^, ^4^, ^5^ Some commentators have argued that is one of the reasons for the inconsistency of results from AESs.^2^ Extension advice on the application of measures has typically been top‐down knowledge transfer. Information from scientists is available to farmers, but often from third parties in a limited and inaccessible format that does not engender trust in practical application and efficacy.^6^, ^7^, ^8^ In addition to this, educational content within AES communication focusses on the practical aspects of how to integrate measures into farming systems, crucially missing the contextual element of why and how the measures work to increase biodiversity and benefit ecosystem functioning and sustainability of farming.^2^, ^9^ Extension bodies that are trusted by farmers can do more to capture hearts and minds, as has been shown to be the case particularly for farmland birds.^10^, ^11^, ^12^

**Figure 1:**
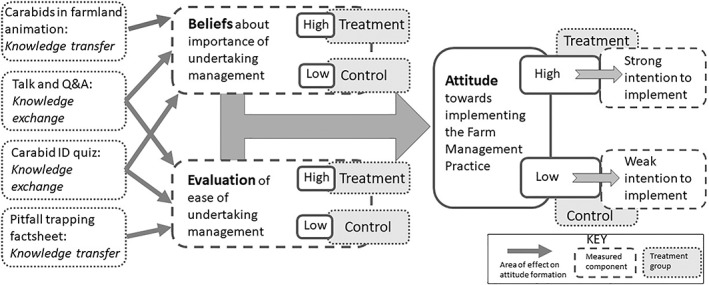
Hypothesised treatment effects, incorporating attitude formation as posited by the Theory of Planned Behaviour. The engagement interventions (left‐hand boxes) were expected to impact beliefs about farm management practices, resulting in higher positive attitudes compared to a control group not receiving interventions, and a stronger intention to implement the practice. Q&A, question and answer.

The main focus of AES extension has tended to address external factors, such as financial needs and technical abilities.^10^, ^11^ Influencing attitudes, therefore, may be one of the missing ingredients of extension when seeking to increase the uptake of IPM. In this regard, Ajzen's^13^ Theory of Planned Behaviour (TPB) has proven to be a viable predictor of farmer behaviours, and is the dominant theoretical basis utilised in the field.^14^, ^15^ The TPB posits *attitudes* as resulting from *beliefs*, multiplied by the *evaluation* of those beliefs.^13^ Both knowledge about the theoretical basis of management interventions and belief in its importance and efficacy are necessary to build the behavioural intent to implement measures in the face of uncertainty (Fig. 1). Knowledge *transfer* alone may therefore not have a strong effect on attitudes, as it has a weak effect on belief evaluation. A growing body of literature supports knowledge *exchange* as a way forward in building attitudes conducive to uptake of agri‐environmental measures, acting on perceptions of efficacy.^16^, ^17^, ^18^ In the agricultural sphere this may comprise schemes for farmer education and farmer groups operating at a local scale and trialling AES design.^9^, ^19^, ^20^, ^21^ Efficacy is also largely interpreted in terms of biodiversity conservation *per se* as opposed to the potential contribution the enhancement of beneficial invertebrates to crop production in the context of integrated pest management (IPM).^22^ As yet, practical application of this is piecemeal.^23^ Here we examine the case of natural‐enemy pest control (NPC), whereby engagement and knowledge exchange have the potential to result in improved IPM through greater uptake and implementation of AES options that are effective for conserving carabids.

### 1.1 Carabids as beneficial organisms in agro‐ecosystems

Over‐reliance on chemical crop protection products (CCPPs) has resulted in negative unintended consequences such as impacts on nontarget organisms and pollution of water courses. This has led policy makers to support more sustainable alternatives to controlling pests, weeds and diseases. The concept of IPM, which aims to integrate nonchemical approaches with pesticides to reduce the reliance of CCPPs, is central to the new approach. Eight principles of IPM have been identified,^24^ one being the prevention and suppression of pests by the protection and enhancement of beneficial organisms. This includes the management of crops and surrounding semi‐natural habitats to build up populations of natural enemies of pests, elsewhere termed ‘conservation biocontrol'.^24^ The increased implementation of IPM by farmers is now explicitly acknowledged as a policy goal at both the European and UK levels.^25^ The UK government^26^ recently published its 25 Year Environment Plan within which it states that ‘We should put Integrated Pest Management (IPM) at the heart of an in‐the‐round approach, using pesticides more judiciously and supplementing them with improved crop husbandry and the use of natural predators.’ Barriers currently exist to meeting this goal. Some are to do with a lack of scientific understanding of the response of beneficials and pests to habitat management, and others are socio‐economic, such as the lack of appropriate advisory support. Reducing the reliance of pesticides using an IPM approach means both equipping farmers with the required knowledge and convincing them of its efficacy. In particular, increasing the uptake of NPC is particularly challenging as the natural control agents are often cryptic and not easily observed. In this paper, we explore the potential for overcoming barriers to take up NPC, specifically by influencing farmers' attitudes to IPM, using the example of carabid beetles.

Carabid beetles have been comprehensively shown to be effective NPC agents, and much is known of the ecology and utility of their ecosystem services in agriculture.^27^, ^28^, ^29^ The impact of management on carabids has also variously been described, including impacts of machinery operations, fertiliser inputs, pesticide effects and habitat provision.^30^, ^31^, ^32^ Decades of carabid research would seem to have covered all the bases to inform practice. However, practice is still substantially lagging behind theory. Despite the documented utility of carabids in relation to crop protection and growing demand for sustainable solutions to pest management,^33^, ^34^ carabid beetles are not widely considered in farm management planning. This is in contrast to more charismatic taxa such as farmland birds that may be less cryptic but have a lesser functional role in supporting crop production.

### 1.2 The disconnect of science and application

Many AES options are potentially beneficial to carabid beetles. Measures such as tussocky grass margins, beetle banks and hedges provide stable resources for carabids between the disturbed habitats that crop areas constitute.^32^ Studies have confirmed that these areas encourage abundance and diversity of carabid beetles that can ‘spill over’ into crop areas.^35^, ^36^, ^37^ However, there is no mention in the current AES programme design or documents given to farmers^1^, ^9^ of their value as agents of pest and weed seed regulation.

Studies have shown that mentioning specific taxa (farmland birds and pollinators) and options targeted at their ecological requirements in AES can often be effective for their conservation.^10^, ^11^, ^38^ Carabids, as a suite of pest and weed seed predators, are vital to the productivity of most farming systems.^33^, ^34^ Yet the only mention of beetles, and the justification for inclusion of beetle banks in AES, is as food resources for farmland birds,^1^ and there is potential to enhance the value of this and other AES options by also considering their role in NCP. Explicitly linking the conservation of biodiversity with its functionality in supporting crop production is also a necessary step to deliver the stated UK policy goal of increasing the uptake of IPM.^26^, ^27^, ^39^

### 1.3 Communicating carabids

Our aim was to identify the key factors that determine the likelihood of farmers implementing management strategies for improved NPC by carabids and to assess their willingness to monitor the impact of management interventions. To that end, we framed our methodology around the antecedents of behaviour as defined in the TPB as beliefs and subsequent attitudes.^13^ We tested for evidence that if an intervention was *believed* to be straightforward to implement and *believed* to have benefit in terms of crop protection (in terms of the farmer's perceptions), it was more likely to be adopted (Fig. 1). In support of this, we designed a questionnaire (The Beneficial Beetles Survey) to measure current awareness of the role carabids play in NPC and the farm management practices (FMPs) that may increase their numbers. To investigate how likely farmers were to uptake FMPs we asked more general questions about the interventions they had previously adopted in support of sustainable production, whether these were done through AES or voluntarily and how difficult farmers perceived each was to implement.

We hypothesised that knowledge exchange would have significant positive impact on farmers’ attitudes to (i) the role carabids play in natural crop protection, (ii) their understanding of the importance of certain FMPs for enhancing NPC and (iii) their perceptions of how difficult implementing certain FMPs might be. To test this, we applied our questionnaire to two groups. A control group, who completed the questionnaire with no known prior interaction with the research team, and an intervention group, who prior to completing the questionnaire undertook an ‘engagement intervention’. For this we designed several resources, including a short educational video, to give an overview of how to conserve carabids in farmland and why it is important, a carabid identification (ID) quiz to build self‐efficacy and familiarity with carabid species, and a factsheet to build self‐efficacy in monitoring carabids (see Supporting Information: Engagement materials).

Our expectation was that the knowledge transfer and knowledge exchange interventions in the treatment group will act strongly on beliefs and evaluations, leading to a higher willingness to implement measures to support carabids.^13^

## 2 METHODS

An online questionnaire was utilised to measure knowledge and beliefs about carabid beetles, their role in NCP and the efficacy of FMPs to conserve them. We used contingency tables to analyse quantitative survey questions, and analysed the frequency and content of qualitative survey questions. We also analysed the attitude constructs from questions on farmer beliefs with general linear models (GLMs).

The questionnaire was disseminated in two rounds. For the first round, participants were not subjected to the knowledge exchange treatment, and we view this as our *control group*. In the second round we also deployed a knowledge exchange treatment (see below). In the first round (April 2020 to June 2020), farmer participants were enlisted in a snowball method through requests included in articles, podcasts, newsletters and social media communications of researcher, institute and agricultural organisations (see Supporting Information). The survey was disseminated by a link hosted in the enlistment communications. We were dependent on voluntary responses to an open request and were constrained by the numbers of respondents. Although providing sufficient power for the control/treatment comparison, an *a‐priori* selection based on controlling factors such as gender, educational background and experience was not possible. However, these factors were captured in the questionnaire and potential effects on the results included in the statistical analysis.

The questionnaire started with an opening statement explaining that carabid beetles are known to play a role in NPC predating on weeds and insect pests. This statement was given as context, with no indication of the significance of the predation. No additional educational information was given to this control group. A review of the existing extension material available to farmers on habitat creation for NPC highlighted the paucity of information on habitat requirements of carabids and their potential contribution to pest control at the level delivered by our new material. We were therefore confident that the control group was not biased by previous access to equivalent educational material.

For the second‐round (June 2020 to September 2020) participants were enlisted through four online events, as well as promotion on social media and relevant agricultural media and newsletters. At each event, there was a talk about carabids in farmland and a question‐answer session, and farmers were given details to take part in the study. Participants in the second round, who we refer to as the *treatment group*, were asked to view engagement material (see Section 2.2 for details) before completing the questionnaire. The treatment group questionnaire was kept separate by closing the control questionnaire, but all questions remained the same, with the exception of a verification question ensuring participants had viewed all educational materials prior to the questionnaire.

### 2.1 Online questionnaire

The questionnaire was split into three sections. Following a context statement about carabids and pest control, the first section measured awareness of carabids and their importance for NPC. In this section we also measured farmers’ belief in their ability to identify carabids, and in the importance of carabids for pest control (see S1: Table S2 for details). At no point did we objectively assess the skill or ability of farmers to identify carabids, rather we designed the questionnaire to understand their belief in their ability and the extent to which this affected their attitudes to managing carabids for NPC.

The second section focused on options for enhancing carabid‐mediated NPC on farmland. We based our questions around 16 FMPs identified in the literature to have effects on carabids (Table 1). The practices can be divided into the provision of suitable noncropped habitat (for which farmers can receive an AES subsidy) and changes in crop management that could also be part of an IPM strategy.^1^, ^24^ To measure experience and implementation, we gathered data on current and past FMPs. To examine motivations, we also asked whether these were undertaken voluntarily or under subsidised AESs, and if they carried out any of the practices specifically for carabids. We measured behavioural intent by asking whether participants would consider carrying out or increasing the amount of the FMPs they carry out in order to benefit carabids. We also asked about the barriers to implementing any of the FMPs. Respondents were asked how important they considered each FMP to be to sustainable pest control mediated by carabids, and they were asked how difficult they perceived undertaking the measures to be (both on a 7‐point likert scale, see Table 2).

**Table 1:** A summary of the questions asked in the questionnaire. The questions that were expected to be influenced by the intervention in the treatment group are indicated by *. See Supporting Information for full content

Question description	Response type
Section 1: Carabids	
Q1: Before today were you aware that the beetles inhabiting your agricultural fields included carabid beetles?	Tickbox response, one could be selected of *Yes* or *No*
Q2: Do you believe you could identify a carabid beetle?	Tickbox response, one could be selected of (i) *Yes ‐ many species*, (ii) *Yes‐ a few species and families*, (iii) *Yes ‐ as distinct from other types of beetle*, (iv) *Not sure*, (v) *Probably not*, (vi) *Definitely not*
Q3a: Before today were you aware that carabid beetles eat crop pests such as aphids, slugs, caterpillars, grubs and mites?	Tickbox response, one could be selected of *Yes* or *No*
Q3b: Before today were you aware that carabid beetles eat crop weed seeds such as dandelion, shepherd's purse and chickweed?	Tickbox response, one could be selected of *Yes* or *No*
Q4a*: Do you believe that carabid beetles can make a significant contribution to crop insect pest control?	Tickbox response, one could be selected of *Yes, No*, or *Not sure*
Q4b*: Do you believe that carabid beetles can make a significant contribution to crop weed control?	Tickbox response, one could be selected of *Yes, No*, or *Not sure*
Section 2: The farm environment and conservation	
Q5: Have you implemented the following farm management? (AESs = agri‐environment schemes)	The response was in the form of a table with rows associated with the FMPs listed in Table 2 and the columns associated with the responses (i) *In the past, through AESs*, (ii) *In the past, voluntarily*, (iii) *Currently, through AESs*, (iv) *Currently, voluntarily*, (v) *No/Not applicable*. Multiple columns could be selected for each FMP
Q6: Do you carry out any of the above [FMPs] particularly with the aim of increasing the abundance of carabid beetles and their associated natural‐enemy pest control? If so could you indicate which and provide some details.	*Yes* or *No* with qualitative response facilitated by a text entry box
Q7*: Which, if any, of the above options would you consider carrying out, or increasing the amount you do, in order to boost the abundance of carabid beetles and their associated natural‐enemy pest control?	Qualitative response facilitated by a text entry box
Q8*: Is there any reason you would be apprehensive about implementing any of the above options?	Qualitative response facilitated by a text entry box
Q9a*: How important in your opinion is the following FMP to improving the control of crop pests by natural‐enemies such as carabids?	The response was in the form of a table with rows associated with the FMPs listed in Table 2 and the columns associated with the responses (i) *Extremely important*, (ii) *Very important*, (iii) *Moderately important‐Slightly important*, (iv) *Not at all important*, (v) *Not sure*
Q9b: How difficult would you rate the following farm management, in terms of implementing it on your farm (in terms of cost, labour, knowledge, equipment and time)?	The response was in the form of a table with rows associated with the FMPs listed in Table 2 and the columns associated with the responses (i) *Extremely difficult*, (ii) *Moderately difficult*, (iii) *Slightly difficult*, (iv) *Not at all difficult*, (v) *Not sure*, (vi) *Impossible due to soil or landscape constraints*, (vii) *Impossible due to legal or tenancy constraints*
Section 3: Farm and farmer attributes	
Q10: What is your farm type? Please tick the box that most accurately describes your farming enterprise.	Tickbox response, one could be selected of 10 options, from Defra categories^9^: (i) *Dairy*, (ii) *LFA/upland grazing livestock*, (iii) *Lowland grazing livestock*, (iv) *Cereals*, (v) *General cropping*, (vi) *Pigs*, (vii) *Poultry*, (viii) *Mixed*, (ix) *Horticulture* Classified for analysis as cereals, livestock, general cropping and mixed
Q11: What is the size of your farm?	Tickbox response, one could be selected of (i) *Under 20 ha*, (ii) *21 to 50 ha*, (iii) *51–100 ha*, (iv) *101–500 ha*, (v) *Over 500 ha* Classified for analysis as under 50 ha, 50–100 ha, 100–500 ha and over 500 ha
Q12: What are the sources of your farming experience and knowledge? Please tick all that apply (multiple boxes can be checked)	Tickbox response, one could be selected of (i) *Farming background*, *Farm work from childhood/leaving school*, (ii) *College course/further education (agricultural)*, (iii) *University level education (agricultural)*, (iv) *Agricultural industry qualification, e.g. BASIS* Classified for analysis as non‐formal education, formal education and industry qualification
Q13: Do you receive advice on farm management from any of the following? Please tick all that apply (multiple boxes can be checked)	Tickbox response, one could be selected of (i) *Agricultural groups/bodies*, (ii) *Conservation organisations*, (iii) *Governmental organisations*, (iv) *Agronomists/professional advisors*, (v) *Industry representatives*, (vi) *Farm events*/*training*, (vii) *Farmer networks/farming colleagues* Classified for analysis as top‐down advice (i)–(v) and participatory advice (vi) and (vii)

**Table 2:** Farm management practices included in the questionnaire, with literature citing significance to carabid abundance or distribution in farmland

Farm management practice	Description	Reference
Habitat provision on uncropped land		
Hedgerow maintenance	Appropriate trimming or laying of hedgerows	28, 29, 32, 36, 46, 47
Hedgerow establishment	Planting of hedgerows	27, 28, 29, 32, 36, 49
Beetle banks	Within field banks of planted vegetation	32, 36, 49, 50
Field margins/buffer strips	Planted strips or noncultivated areas of grass at edges of fields	28, 29, 32, 45, 46, 55, 56
Ditch maintenance	Clearance of ditches for retention	29, 32, 36, 46, 49
Ponds/wet areas/waterbody creation	Creation of waterbodies or wet areas	28, 29, 32, 49
Fallow land	Land left fallow, without agricultural production, for 1–5 years	32, 36
Natural area retention (e.g. woods, grassland)	Retention of natural unproductive areas such as woodland and grassland	27, 29, 32, 35, 57, 58
Crop management		
Cover cropping	Cropped with a plant primarily to improve soil health within a crop rotation	36, 46, 59
Under sowing/companion crop	Crops with later growing crop sown to grow underneath/different crops grown in proximity	36, 60, 61, 62
Extensive (low) grazing	Livestock system with low density of cattle	28, 63, 64
Low fertiliser input	Low input of fertilisers on land	28, 29, 36, 45, 49
Reduced tillage	Minimum soil manipulation, particularly inversion, in cropping	30, 36, 45, 59, 65, 66, 67
Diverse cropping/rotations	A typical intensive rotation in the UK is dominated by wheat with most intensive cropping systems growing wheat 2 years in thee. More diverse rotations are anticipated to be at least a 5‐year rotation breaking cereals with a mixture of brassicas, legumes and grass leys	36, 49, 68, 69
Low herbicide use	Low use of herbicides for weed control or crop management	46, 70
Low pesticide/antihelminth use	Low use of pesticides for pest control, including wormers in livestock	28, 29, 46, 71

To set the results of the questionnaire in context, and to control for mediating variables, the third section related to questions on basic farmer demographic data. This comprised information on profession, farm typology, farm size, education and sources of advice.

The impacts of the Covid19 outbreak in 2020 created some constraints for the planned work. The main one of these was limited time and resources to carry out a comprehensive pilot of the questionnaire. Therefore, the questionnaire was subject to expert review at both institutes co‐ordinating the study and piloted in qualitative interviews with four farmers from diverse backgrounds. Content was altered according to feedback. The questionnaire took between 20 and 45 min to complete.

### 2.2 Engagement materials

The engagement material made available to the treatment group comprised an interactive talk, an animation, a factsheet and an educational quiz (Supporting Information). The talk was 30 min long, split into three sections: (i) carabid ecology, (ii) farm measures for carabid abundance and diversity, and (iii) how and why to monitor carabids. After each section farmers were given the opportunity to ask questions and make comments. The 3‐min ‘Carabid beetles in farm environments’ animation was designed to communicate key concepts of carabid ecology, including how and why they move in farm landscapes, and highlight their role in pest and weed‐seed control. The factsheet was designed to build self‐efficacy and engage farmers in carabid monitoring. The short ID quiz was designed to engage farmers with carabid ID and teach basic ID skills. Questions were multiple choice with pictures of carabid beetles, followed by explanatory text on ID techniques. Participants for the treatment group were recruited from three 1‐h events where the talk was given (Table S1). Participants were emailed materials and an ethics statement.

### 2.3 Statistical analysis

Due to the impacts of the Covid19 outbreak in 2020, engagement events took place online rather than in person as planned. In all we received 190 responses to the questionnaire, 160 in the control and 30 in the treatment group, which received the engagement materials. Although we sent reminder requests to online talk participants we were reliant on self‐selection and thus the treatment group was smaller than we anticipated, but large enough for valid statistical comparison.^40^ We chose to exclude responses where the first two questions were not completed, leaving 138 responses. For analysis of Section 2 of the questionnaire (farm environment and conservation measures), we further excluded responses where less than 80% of this section was answered.

For the questions in Section 1 of the questionnaire (Table 1), to account for mediating variables, we first tested to see if there were significant differences in responses according to demographic data (farm type, farm size). To do this we constructed contingency tables where the columns of the table related to the demographic class (e.g. in the case of farm type, the columns were the farm classification) and the rows the responses to the question ask (e.g. for Q1, the rows related to ‘yes’ and ‘no’). The categories for farmer demographic (farm type, size, background and source of advice) were relatively detailed. To avoid categories with too few responses we aggregated to coarser scale categories (coarse scale categories are shown in Table 1).

Under the null hypothesis responses are independent of demographic type and so the same distribution of responses is expected. That is to say, the expected number of responses in a cell is the product of the respective marginal (row and column) totals divided by the total number of responses in the table. If the expected number of responses in the i th cell (out of N) is $e_{i}$ and the observed number is $o_{i}$, we then compute a statistic to measure the evidence against the null hypothesis. In principle under the null hypothesis, and with $n_{r}$ rows and $n_{c}$ columns in the table

$$X^{2}=\sum_{i=1}^{N}{\left(o_{i}-e_{i}\right)}^{2}/e_{i}$$

is distributed by $\chi^{2}$ with $\left(n_{c}-1\right)\left(n_{r}-1\right)$ degrees of freedom, but the fact that $o_{i}$ is an integer introduces an approximation when the $o_{i}$ over many cells is small. For this reason, we obtain a *P* value for $X^{2}$ under the null hypothesis by the permutation method.^45^ In the event, we found no significant differences according to farmer demographics and so we did not test for these differences in relation to the responses for questions in Sections 2 and 3 (which were more complex in structure).

To test our hypothesis that engagement with farmers would have a positive impact on awareness, beliefs and perceptions of FMPs to enhance natural‐enemy IPM, we used the $\chi^{2}$ permutation test to determine whether there were significant differences in responses between control and treatment groups for questions indicated by * in Table 1. To analyse Q5, we also used the $\chi^{2}$ permutation test to determine whether there were significant differences in the types of FMP undertaken voluntarily compared with AESs both now and in the past (Q5 from Section 2). We also pooled responses over AESs and voluntary for the two time periods and used a $\chi^{2}$ permutation test to test for significant differences in the FMPs adopted by farmers between the two time periods. Qualitative comments (Q6–Q8) were categorised according to whether they mentioned particular practices or not. We were particularly interested in the types of FMP that farmers implemented with the aim of increasing the abundance of carabid beetles and their associated natural‐enemy pest control (Q6), and which they might consider implementing for this reason in the future (Q7) and how this might be impacted by our treatment. For Q9 we also used the $\chi^{2}$ permutation test to determine whether there were significant differences according to management type.

Under the TPB, attitudes are a product of beliefs multiplied by evaluations.^13^ To visualise Q9a and Q9b (see Table 1) under this framework, we calculated the average ‘belief’ in first the importance (Q9a) and secondly the difficulty of implementation (Q9b) for each FMP by applying numerical scoring to the categories and plotted them together. We scored ‘Extremely important’ as 4, through lowering importance, down to 0 for ‘Not at all important’, and ‘Not at all difficult’ as 4, down to 1 for ‘Extremely difficult’. We excluded categories of ‘Impossible’ as outside of theoretical decision making and scored ‘Not sure’ as median.^42^, ^43^

To determine to what extent the probability of an implementation of an FMP for NPC accorded was determined by these beliefs, responses to Q6 and Q7 (FMPs that farmers are currently doing or would consider doing in the future) were modelled using data on belief in the importance of an FMP (Q9a) and difficulty of application (Q9b) as explanatory variables. We took the categorised responses to Q6 and Q7 and assigned one for mentioning or zero for not mentioning each FMP. Responses indicating that the participant did not practice any FMPs for carabids (Q6) or intended to do so (Q7) were excluded. We fitted general linear models (GLMs) using the Genstat statistical software package^41^ to determine the effect of the perceived importance of FMPs (Q9a) and the perceived difficulty of FMPs (Q9b) on the response variables quantified from Q6 and Q7. This included treatment and control groups as a factor to test our main hypothesis. We modelled only participants answering Q9. We excluded those answering ‘impossible’ to Q9 as these cannot be said to be making a decision, and ‘not sure’ for both questions as these cannot fit into an ordinal scale of perception. We assumed a binomial distribution, and considered the importance, difficulty and treatment level factors as fixed effects with three‐way interactions. We selected terms using backwards elimination according to the largest *P* value given by the Kenward–Roger approximate *F*‐tests.^40^ The final predictive model was chosen when all remaining terms gave significant values (*P* ≤ 0.05) when dropped from the model.

## 3 RESULTS

### 3.1 Summary of data

For the control questionnaire 116 responses contained enough data for analysis. The subset of full responses to Section 2 of the questionnaire comprised 66 responses. Qualitative answers were collected from 67 responses. For the treatment questionnaire 22 responses contained enough data for analysis. The subset of responses to Section 2 of the questionnaire responses comprised 19 responses, all of which included qualitative responses.

There were no significant differences in farmer demographics between treatments (Table 3). The majority of participants were arable farmers (cereal crops and general cropping). A large proportion had mixed farms, and a much lower proportion farmed livestock alone. The smallest proportion comprised horticulture. The majority of participants reported farm size of 101–500 ha, followed by larger farms of >500 ha. The smallest proportion of respondents had farms less than 20 ha in size. The demographics of our participants varied from national averages^9^ in a larger median farm size and a greater proportion of cereal farmers.

**Table 3:** Farm and farmer demographics by treatment group

Q10: Farm type								
Group	Cereals	Dairy	General cropping	Horticulture	LFA/upland livestock	Lowland livestock	Mixed	Total
Control	23 *36.5%*	2 *3.2%*	10 *15.9%*	3 *4.8%*	0	4 *6.3%*	21 *33.3%*	63
Treatment	5 *26.3%*	0	5 *26.3%*	0	1 *5.3%*	0	8 *42.1%*	19
Both	28 *34.1%*	2 *2.4%*	15 *18.3%*	3 *3.7%*	1 *1.2%*	4 *4.9%*	29 *35.4%*	82
Q11: Farm size (hectares)								
Group	under 20	21–50	51–100		101–500	Over 500		Total
Control	1 *1.6%*	4 *6.5%*	8 *12.9%*		34 *54.8%*	15 *24.2%*		62
Treatment	1 *5.3%*	0	0		16 *84.2%*	2 *10.5%*		19
Both	2 *2.5%*	4 *4.9%*	8 *9.9%*		50 *61.7%*	17 *21.0%*		81
Q12: Sources of knowledge or experience								
Group	Conservation groups	Governmental	Agricultural groups	Agronomist	Industry representative	Events/training	Farmer networks	Total
Control	39 *60.9%*	24 *37.5%*	39 *60.9%*	46 *71.8%*	22 *34.4%*	46 *71.9%*	45 *70.3%*	64
Treatment	14 *73.7%*	9 *47.5%*	16 *84.2%*	14 *73.7%*	7 *36.8%*	18 *94.7%*	17 *89.5%*	19
Both	53 *63.9%*	33 *39.8%*	55 *66.3%*	60 *72.3%*	29 *34.9%*	64 *77.1%*	62 *74.7%*	83
Q13: Sources of farm advice								
Group	Farming background	Farming from childhood	College		Industry qualifications	University		Total
Control	53 *23.1%*	31 *15.4%*	27 *20.0%*		25 *10.7%*	22 *4.6%*		65
Treatment	15 *78.9%*	10 *52.6%*	13 *68.4%*		7 *36.8%*	3 *15.8%*		19
Both	68 *81.0%*	41 *48.8%*	40 *47.6%*		32 *38.1%*	25 *29.7%*		84

Percentages of group total response in italics. Q12 and Q13 respondents could choose multiple categories.

Participants could select multiple sources of knowledge and experience (Table 3). A ‘farming background’ and farming ‘from childhood’ were most frequently selected. Formal education was most frequently selected as college, followed by industry qualifications, then university. Similarly, multiple sources of advice could be selected (Table 3). Most frequent were events and training, farmer networks, agronomists, agricultural groups and conservation groups. Less frequently selected was governmental advice and industry representatives.

### 3.2 Section 1: Awareness and beliefs about carabid‐mediated natural‐enemy pest control

For the four awareness questions (Q1–Q4), there were no significant effects of treatment group or the demographic groups (farm type, size) on the responses, therefore we pooled the data across typologies and treatments. Of the 138 respondents, 87.0% were aware of carabid beetles before participation (Q1). One third indicated that they could identify a carabid beetle as distinct from other beetles, whilst 30.4% were unsure (Q2). Responses of confidence in identifying many species and responses that they could not identify carabids at all shared the lowest frequency, both at 4.3%. Although 80.4% of respondents were aware before participation in the questionnaire that carabid beetles ate crop pests, only 25.9% were aware that carabid beetles eat weed seeds (Q3). Similarly, 77.5% of respondents believed that carabids could make a significant contribution to crop pest control and only 2.9% did not believe as such, with a further 19.6% unsure, whilst only 29.6% believed that carabids could make a significant contribution to weed control, 16.2% did not believe as such, with the largest proportion at 54.0% unsure (Q4). There was no significant difference in the responses to Q4a and b according to treatment.

### 3.3 Section 2: Farm environment and conservation

**Figure 2:**
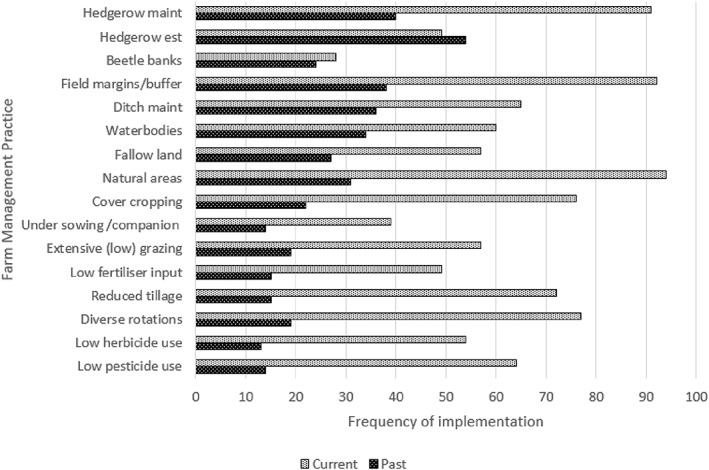
Farm management practices implemented by all participants (control and treatment) in the past and currently (Q5).

Answers to Q5 showed that most respondents had adopted one of the FMPs listed. The most frequently selected was margins/buffer strips, followed by hedgerow maintenance, natural area retention, diverse rotations and reduced tillage. The least selected were beetle banks, fallow land and undersow/companion crop (Fig. 2).

There was a significant difference between past and current implementation (*P* < 0.001, $X^{2}$ 62.40, 15 df), and overall there has been an increase in implementation of the FMPs (Fig. 2). There were also significant differences in the types of FMPs adopted. Hedgerow establishment and beetle banks were more frequently adopted in the past, and reduced tillage more frequently adopted currently than would be expected under the null hypothesis. Of the past implementation, there was a highly significant difference in FMPs adopted voluntarily or through AES (*P* < 0.001, $X^{2}$ 61.26, 30 df). Reduced tillage and diverse crop rotation were adopted more voluntarily, whilst beetle banks and field margins/buffers were adopted more under AES than would be expected under the null hypothesis.

Of the current implementation, there was a significant difference between voluntary and AES implementation (*P* < 0.001, $X^{2}$ 153.10, 30 df). Reduced tillage, diverse rotation and low pesticide use were adopted voluntarily more than expected under the null hypothesis, whereas the adoption of margins/buffer strips, both hedgerow establishment and maintenance, and beetle banks was less than expected. The difference between the FMPs adopted voluntarily in the past was significantly different from that adopted currently (*P* = 0.006, $X^{2}$ 52.76, 30 df) and the difference between past and current implementation by AES was not significant (*P* = 0.953, $X^{2}$ 18.31, 30 df).

There were 72 qualitative responses to Q6. Given that this question relates to past activities we pooled the responses for analysis. Overall, 66% of responses indicated that they currently carry out FMPs for carabids. The FMP most frequently mentioned for carabids was reduced insecticide use (30.0%), followed by beetle banks (15.0%) and reduced tillage (12.0%). In further comments, the general value of invertebrates or ecosystem function was mentioned in 18% of responses, with pollinators specifically in 4%, for example ‘Main aim is to increase abundance of ALL insects, carabids, pollinators and other predatory species alike’. A further 8% specifically mentioned soil health, for example ‘We are actively cover cropping and moving to zero tillage to promote all aspects of soil health including being a positive contributor to the insect world’.

For Q7, there were 73 qualitative responses. This question relates to the future intent of participants and so we expected to see a difference between the groups. For the control group 89% and treatment group 100% indicated that they would consider carrying out or increasing FMPs for carabids. For both groups, the FMP most frequently mentioned with intention to implement or increase implementation was margins/buffer strips (16.9%), followed by beetle banks (13.3%), then cover crops (12.0) and reduced tillage (10.8%). Notably, 12.5% of the control group indicated they would consider reducing insecticides, whilst no one from the treatment group specifically mentioned this. The most frequent comment (control 26.3%, treatment 18.7%) was that they would consider *all* of the FMPs, for example ‘Any of them if I understand what they do and the benefits’. In further comments 3.1% of the control group and 10.5% of treatment group indicated the need for further advice, for example ‘I would like an advisor to visit to see what would be best for the farm as my knowledge is limited’. In 7.8% of the control and 5.3% of treatment responses, participants stated that they already do all or nearly all they can, for example ‘as it is an organic farm much of this is done anyway’. For the control 10.9% indicated a need for AES support, with 4.5% specifically mentioning financial constraints, a further 1.6% mentioned potential loss of productivity, for example ‘depending on finances and schemes available’.

There were 79 qualitative responses for Q8, which asked about apprehension around implementation. For both groups, nearly 60% indicated that there was a reason they would be apprehensive. For both groups, financial constraints were the most cited, followed by loss of productive land and the potential for weed incursion into crops, for example ’…have a large influence on yield and therefore financial return’. Time effectiveness, risk of crop loss and crop quality concerns were mentioned less often, along with physical constraints such as drainage.

**Figure 3:**
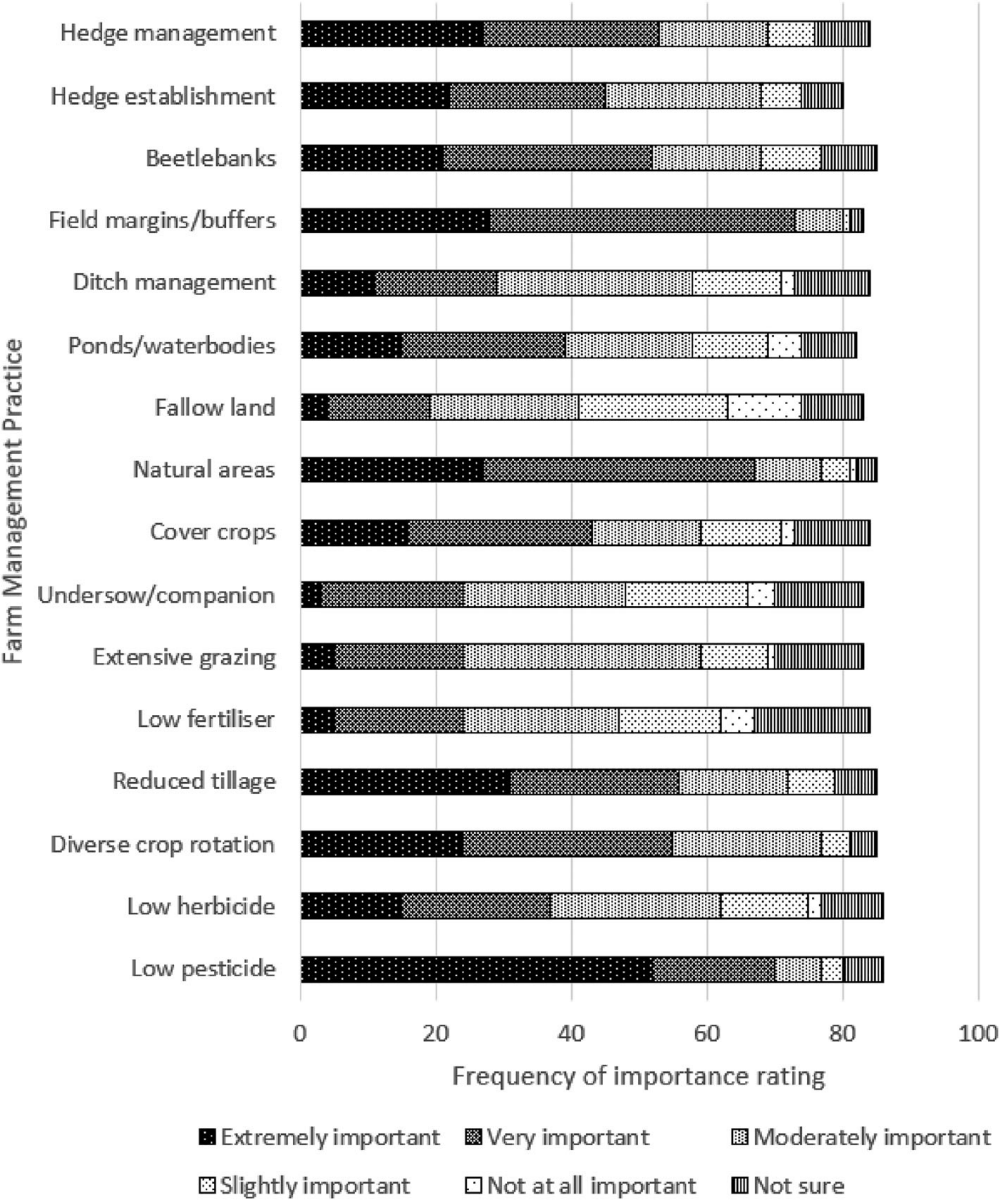
Farm management practices and perceptions of importance for carabids, as rated in responses to Q9a.

For Q9a, on the importance of FMPs for crop pest control by natural enemies such as carabids, the FMP most frequently ranked as ‘Extremely important’ was low pesticide use, followed by reduced tillage, margins/buffer strips and natural area retention. The FMP most frequently ranked as ‘Not at all important’ was fallow land, followed by low fertiliser use (Fig. 3). There were no significant differences between treatment groups.

**Figure 4:**
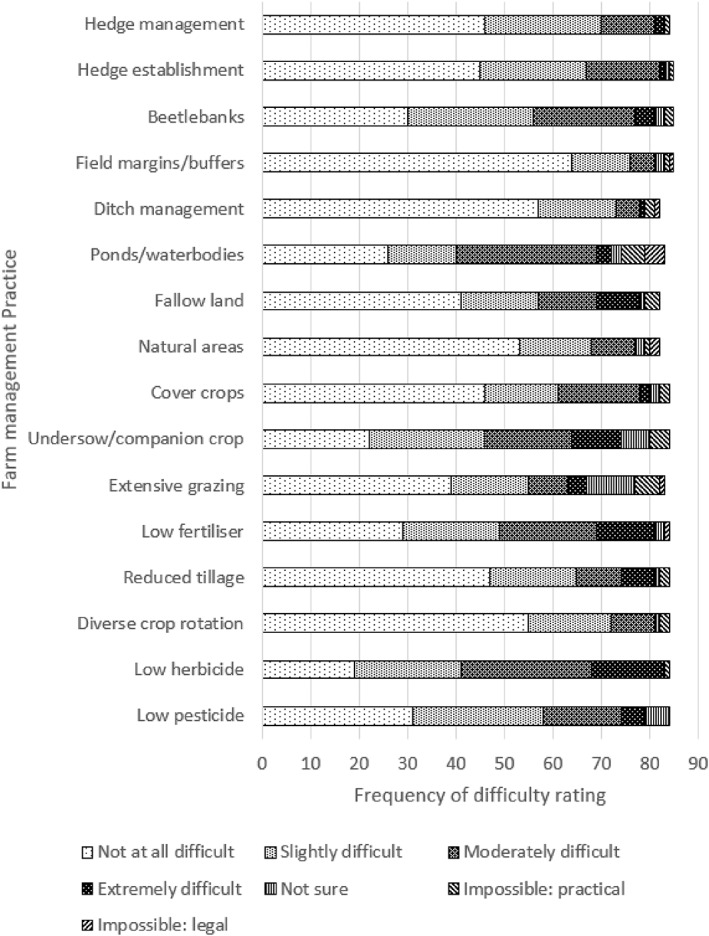
Farm management practices and perceptions of difficulty to implement, as rated in responses to Q9b.

For Q9b, on the perceived difficulty of implementation, the FMP most frequently ranked as ‘Not at all difficult’ was margins/buffer strips, followed by ditch maintenance, diverse rotation and natural area retention. Low herbicide use and low fertiliser use were most frequently ranked as ‘Extremely difficult’. Ponds/waterbodies and low herbicide use were most frequently ranked as ‘Moderately difficult', and low pesticide use and beetle banks were most frequently ranked as ‘Slightly difficult’ (Fig. 4). There were no significant differences between treatment groups.

**Figure 5:**
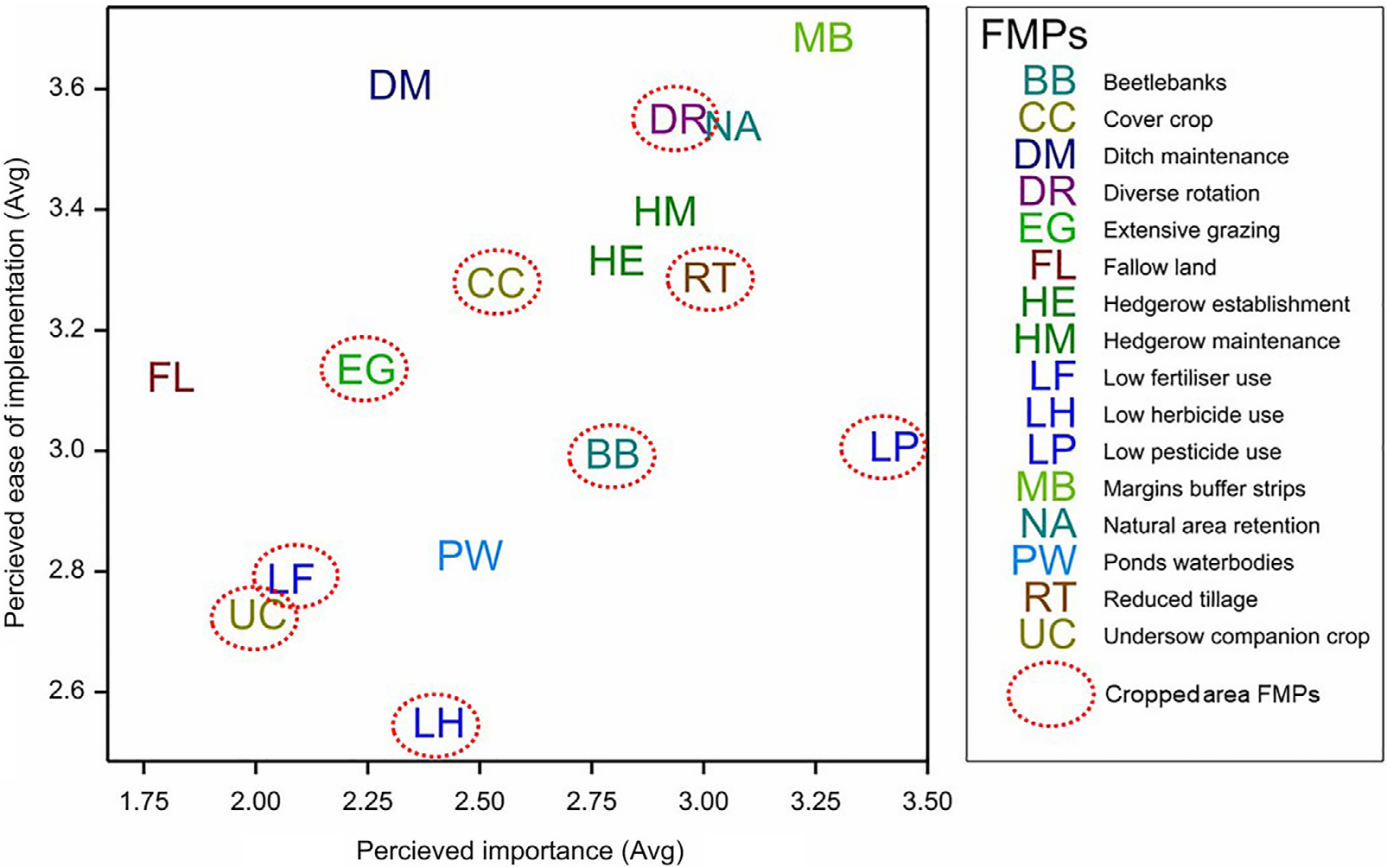
Average scores for Q9a Perceived importance of farm management practice (FMP) for carabids and Q9b Perceived difficulty of implementing the FMP.

To visualise Q9a and Q9b under the TPB framework, scored responses were plotted together (Fig. 5). Margins/buffer strips scored highest for both importance and ease, followed by diverse rotation and natural area retention. Ditch maintenance scored highly for ease yet low for importance, and conversely low pesticide use scored high for importance and lower for ease. Undersow/companion crop and low fertiliser use scored low for both importance and ease.

**Figure 6:**
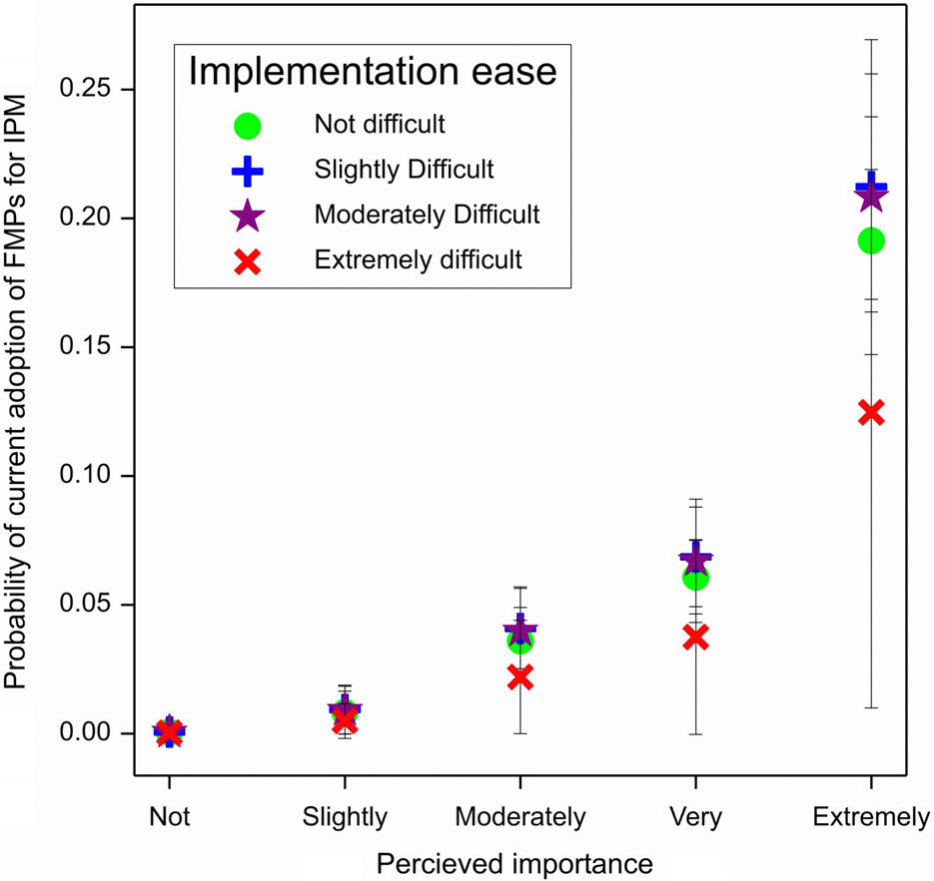
Model predictions for the Theory of Planned Behaviour framework. Q6 Current implementation of farm management practices (FMPs) for integrated pest control by carabids, with perceived importance of FMP (Q9a) and perceived ease of FMP (Q9b).

The fitted GLMM model for current implementation of FMPs for carabids retained both difficulty and importance (7 df, *F* = 13.82, *P* < 0.001). Treatment was not retained in the model and there were no interaction effects (Fig. 6).

**Figure 7:**
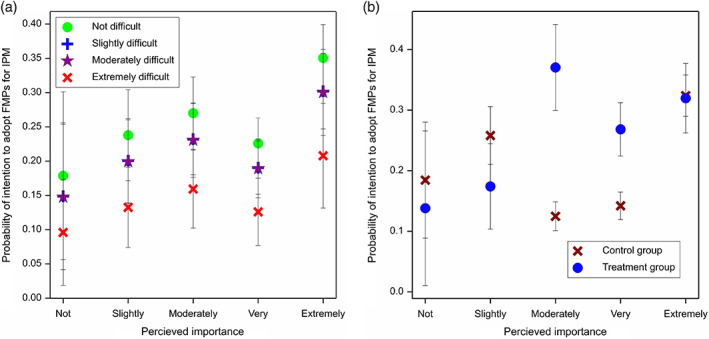
Model predictions for the Theory of Planned Behaviour framework. Q7 Future intent to implement farm management practices (FMPs) for integrated pest control by carabids, with (a) perceived importance of FMP and perceived ease of FMP and (b) control group and treatment group.

The fitted model for intention to implement FMPs for carabids retained all terms of treatment, difficulty and importance (Fig. 7(a)), with an interaction of importance and treatment (Fig. 7(b)) (12 df, *F* = 3.51, *P* = 0.007).

## 4 DISCUSSION

### 4.1 The theory of planned behaviour and UK farmer decision making

In this study we aimed to elucidate the key factors influencing the implementation of FMPs for IPM by carabids, using a theoretical framework based on Ajzen's^13^ TPB. The TPB is often used with expanded conceptual aspects to suit the context of belief formation.^13^, ^14^, ^15^ The results show that the TPB is a useful framework when considering the factors surrounding implementation. First, we see that those FMPs generally perceived to be both highly important for carabids and easy to implement were the ones that had already had the highest uptake. The responses to Q5 (past and current implementation) revealed that the most frequently adopted FMPs were margins/buffer strips, hedgerow maintenance, natural area retention, diverse rotations and reduced tillage, and the plot of average scores (Fig. 5) shows these particular FMPs clustered around the top right corner, where we would expect to see practices that are likely to be adopted under the TPB.

Some interesting nuances are apparent. Low pesticide use is ranked as very important for carabids yet somewhat difficult to implement, and this was in the median of FMPs adopted. Other in‐field options, including reduced herbicide use, fertiliser use and companion cropping, were also perceived as being difficult to implement but of less importance for carabids. However, we note that Q5 asked only what FMPs had been adopted, not those adopted specifically for carabids, although Q6 (implementation for carabids) further reveals that the FMP most commonly adopted for natural‐enemy pest control (NPC) was low pesticide use. Ditch maintenance was perceived as very easy, yet not ranked as very important for carabids (Fig. 5). Similarly, the discrepancy of the least adopted FMP according to Q5 being beetle banks, despite its central position on the plot of perceived importance × perceived ease of implementation, may be attributed to the fact that it was carried out specifically for the benefit of natural enemies, including carabids, as seen in frequent comments for Q6.

The model explaining uptake of FMP further confirmed the TPB framework, with significant terms of both perceived importance and difficulty of implementation explaining which FMPs were adopted specifically for natural‐enemy pest control. Treatment proved not to be a significant factor in the model, and this is to be expected as it could not have affected the decisions already made (i.e. current management). Regarding future intention to implement management for carabids as evidenced by Q7, the model‐based analysis revealed that importance and difficulty were again significant (Fig. 5), but this time treatment was also retained in the model (Fig. 7). This supports our hypothesis in that the treatment had an effect on the strength of future intent to implement FMPs for carabids. This result also demonstrated the potential to encourage uptake of specific FMPs by influencing farmer perceptions about the efficacy of NPC. It also provides evidence for the importance of evaluation: shared experience of the successful implementation and efficacy of a current FMP is likely to encourage increased uptake in the future.

Fig. 5 shows the treatment had the largest effect in relation to participants responding with rankings of ‘moderately’ and ‘very important’, shifting the probability of adoption higher in the treatment group compared to the control group. This may be attributed to the top portion being already persuaded, as the TPB hypothesises that strong beliefs of importance alone can lead to adoption, despite difficulty.^13^ This is supported in qualitative responses to Q7 where some participants felt they already did all they could. The Elaboration Likelihood Model^44^ suggests that beliefs of importance create stronger attitude change through higher engagement with persuasive materials. A high motivation causes receivers of a message to cognitively appraise the message content, whilst low motivation in receivers results in less scrutiny of the message.^44^ This may explain the responses of ‘not important’ and ‘slightly important’ being less influenced by the treatment content, for example by cognitive dismissal of FMPs mentioned in the talks that were perceived as unimportant. These results could be used to target knowledge exchange activities at FMPs for which there is most potential to influence farmer behaviour.

### 4.2 Targeting of FMPs for outcomes

The most favoured FMP in respect to future intent was margin/buffer strips, which accords with Fig. 5 and the TPB. Field margins and buffer strips have been comprehensively shown to be beneficial habitats for carabids, providing hibernation, aestivation and stable resources in proximity to crop areas prone to disturbance.^29^, ^32^, ^45^ However, margins are not necessary for all carabid species of significance to IPM and moreover spill‐over into crop habitats for pest and weed control is not guaranteed.^50^ Other FMPs may be more desirable to boost abundance of beneficial species for IPM.

Butler et al.^47^ examined the uptake of FMPs for cropped and noncropped areas and found that despite there being more AES options for cropped areas, the main focus of current agreements was on hedgerow and margin management. This accords with our findings (Fig. 5), which largely confirm that interventions in noncropped areas are more favoured. However, diverse rotations and reduced tillage are more popular than expected considering Butler et al.'s work, and this is likely to be because interest in regenerative farming practices has grown since the publication of the Butler study. These options were not widely supported in the past by AES,^48^ yet farmers increasingly deem them of sufficient importance and as having lack of difficulty to implement, which may also reflect importance for other farmer priorities such as soil conservation.

Beetle banks are designed to support beetles and whilst not exclusively aimed at carabids, they provide a range of microclimates and alternative food resources, and are connected to edge habitats, theoretically nudging carabid abundances to field centres for IPM.^32^, ^36^, ^49^, ^50^ Despite the potential benefits for crop pest control we found beetle banks to be the least adopted FMP overall (Q5).

Beetle banks were, however, the second most mentioned FMP as currently implemented for carabid beetles in Q6 and the second most mentioned with future intent in Q7. This may be due to the balance of values in decision making. Farmers are subject to a range of influences on their decision making. IPM by natural enemies is only one facet of a healthy farm environment, and other FMPs may have perceived benefits outweighing the consideration of FMPs for carabids.

### 4.3 Lessons for communicating carabids to increase the uptake of IPM

The questionnaire responses showed that, prior to the survey, most participants were aware of carabids in agricultural fields and their role as predators of crop invertebrate pests. This was reflected in their beliefs about the efficacy of carabids for IPM of invertebrate pests. However, there was much lower awareness of their weed seed predation, likewise reflected in their lower level of belief in the efficacy of this, contrary to the evidence in the literature that weed regulation by carabids is significant.^28^, ^51^

We hypothesised that engagement materials would have the effect of more positive beliefs in efficacy and more willingness to apply FMPs for carabids. The lack of difference for questions of attitude and belief between treatments may have been due to the sample attributes. Participants were self‐selected and as such were likely motivated individuals.^44^ Farmer participants had a higher than average education level, and tended to participate in training and networking to acquire information, rather than relying on advisors alone.^52^, ^53^, ^54^ The overwhelming majority also responded positively to Q7 on intent to apply FMPs for IPM in the future, demonstrating high motivation. The lack of significant differences between demographic variables may likewise be attributed to the homogenous sample.

Figure 1 is a simplified diagram showing only the conceptualised treatment effects on attitudes as a determinate of behavioural intent. In actualised scenarios, decision makers are subject to a range of factors and constraints governing the uptake of FMPs. Financial concerns were raised in qualitative responses, notably as unprompted responses from the control group to Q7 on future intent. Since we see that the most popular measures for IPM by carabids have been adopted more by AESs (Q5), this is important to consider. This factor and the higher biodiversity gain^48^ in cropped area FMPs lead us to propose that more effective financial support, or a demonstration of long‐term financial benefits, for FMPs such as diverse rotations, reduced tillage and low pesticide use (that are not fully supported in AESs), is likely to have a higher impact. In Fig. 5 we show that attitudes are positive towards these FMPs, so targeting practical constraints may bridge the gap between attitudes and adoption.

Given past disconnect between science and application, the high level of general engagement with the survey demonstrates the interest of farmers in beneficial insects for IPM. Attendance at talk events on carabids and qualitative comments demonstrates the desire for information, which further feeds into an argument for better provision of advice to support natural enemy IPM. While our results have provided strong evidence for the potential benefit of targeted farmer engagement in improving the uptake of FMPs that benefit carabids, further work should seek to engage a wider cross‐section of the industry in terms of educational background. Additional engagement work with farmers that includes monitoring of carabids on farms and an assessment of its importance in changing or affirming attitudes would also be beneficial.

## 5 CONCLUSIONS

The TPB is widely used in research around farmer behaviours, yet few studies document agro‐ecological applications of this theory. Our findings confirm the utility of the TPB in examining where interventions may impact farmer decision‐making on FMPs for natural‐enemy pest control (NPC). Online engagement materials were useful in targeting perceptions of the importance of FMPs for NPC and increasing the probability of future adoption of FMPs to benefit carabids for IPM. Perceptions of difficulty of application may be better targeted by practical engagement.

Farmer perceptions about the importance of FMPs in relation to NPC and how difficult these practices are to implement varied. This corresponded to past and current patterns of FMP adoption. Farmers had the highest positive attitudes to margins/buffer strips, hedgerow management and natural area retention. These may be easy wins in terms of take up, but more impactful intervention would target cropped areas, for example diverse crop rotations and reduced tillage. These results highlighted the need for natural scientists to engage with and address socio‐economic barriers to uptake when designing management interventions for IPM.

Farmers participating in this study were engaged by information about carabid beetles and the implementation of IPM principles for sustainable pest control. We saw a level of trust in direct science communication, which is encouraging. We recommend targeted engagement for enhanced uptake of IPM principles. Online materials were effective on farmers with neither very positive or very negative beliefs; more practical interventions may change attitudes and combat negative views on importance. The approach taken here could readily be applied to other components of functional biodiversity linked to farm production (e.g. earthworms) to help inform and motivate farmers to adopt sustainable practices for IPM.

## AUTHOR CONTRIBUTIONS

KJ, AEM and JS conceived and designed the study. The research and analysis were performed by KJ and AEM with input from JS. All authors contributed to interpretation of results and writing the manuscript.

## Supporting information


**Appendix** S1: Supporting information

## Data Availability

The data that support the findings of this study are available on request from the corresponding author. The data are not publicly available due to privacy or ethical restrictions.
