# Supplementary material for: Communicating carabids: Engaging farmers to encourage uptake of integrated pest management
Source: Pest Manag Sci. 2022 Apr 7;78(6):2477–91. doi: 10.1002/ps.6878 (PMC9322692; doi:10.1002/ps.6878)
Supplement: Supplementary file 1 — Appendix S1: Supporting information [file PS-78-2477-s001.docx]

Supplementary

**Participant recruitment**

Requests included in newsletters and social media communications of agricultural organisations: Linking the Environment and Farming (LEAF), Agricology, Biodiversity Agriculture Soil and Environment (BASE), Championing the Farmed Environment (CFE), The Farming and Wildlife Advisory Group (FWAG), Agri-Tech- east, The Agriculture and Horticulture Development Board (ADHB), and the National Farmers Union (NFU).

Articles: in agricultural magazine Practical Farm Ideas [print issued] available from https://www.farmideas.co.uk/, and Farm wildlife UK [online blog] Available at: https://farmwildlife.info/2020/07/12/case-study-carabid-beetles-for-natural-enemy-pest-control/.

Podcasts: Farmers weekly episode 4 available at <https://www.fwi.co.uk/news/farmers-weekly-podcast-episode-4-covid-19-loans-and-red-tractor-inspections> , and Wellies and Labcoats available at <https://soundcloud.com/mandy-stoker-414270483/wellies-and-labcoats-getting-started>

Feature in institutional news story, available at <https://www.rothamsted.ac.uk/news/researcher-makes-internet-appeal-after-covid-19-stymies-her-research>

Presented at the online ‘Cereals’ agricultural show 9-11 June 2020, in the Rothamsted Research site area.

Researcher and institute social media promotion on twitter #BeneficialBeetlesSurvey

**Engagement materials**

Educational video (<https://youtu.be/vNyTzU96yYA> )

Carabid ID quiz (<https://readingagriculture.eu.qualtrics.com/jfe/form/SV_byGTrOfFP9TG2Ud> )

Monitoring factsheet (<https://www.rothamsted.ac.uk/sites/default/files/How%20to%20pitfall%20trap%20on%20your%20farm.pdf> )

Table 1- Online carabids in farmland Talk events conducted as part of the engagement treatment

| Event | Date and time | Access and follow-up |
| --- | --- | --- |
| **Arden Farm Wildlife Network**. Incorporating Warwickshire Rural Hub | Jun 17, 1pm | Attendees by invite of organisers only. Around 40 farmers. Follow-up by email reminders of organisers |
| **BASE farmers talk**  Available to BASE member farmers. Video available on BASE website. | 9^th^ July, 7pm | Attendees by invite of organisers only. Around 30 farmers. Follow-up by email reminders of organisers. Follow-up on fertiliser and pesticide questions on BASE website. |
| **BASIS talk**  Talk with BASIS accreditation points for attendees, organised by Rothamsted. | 14^th^ July, 12.30pm | Attendance open by link promoted on Rothamsted media. Around 30 farmers. Follow-up by email and social media. |

Table 2- Full questionnaire content

| **Question**  **Description** | **Response type** |
| --- | --- |
| ***Section 1 Carabids*** | |
| Statement on carabids: “*Carabids (sometimes called ground beetles) have been shown to be effective predators of crop pests such as aphids, slugs, caterpillars, grubs and mites. They also feed on weed seeds such as dandelion, shepherds purse and chickweed. This type of pest control is termed "natural-enemy pest control".*  *In this survey we are interested in your opinions on natural enemy pest control provided by carabid beetles, and the management of habitats on farms that may promote their abundance.”*  [picture of Pterostichus sp. Showing jaws open] | |
| Filter question for K-E treatment only- verification that participants have viewed all of the materials: animation; quiz; and factsheet | |
| **Q1** Before today were you aware that the beetles inhabiting your agricultural fields included carabid beetles? | Tickbox response, one could be selected of *Yes* or *No* |
| **Q2** Do you believe you could identify a carabid beetle? | Tickbox response, one could be selected of *(i) Yes - many species; (ii) Yes- a few species and families (iii) Yes- as distinct from other types of beetle; (iv) Not sure; (v) Probably not; (vi) Definitely not* |
| **Q3a** Before today were you aware that carabid beetles eat crop pests such as aphids, slugs, caterpillars, grubs and mites? | Tickbox response, one could be selected of *Yes* or *No* |
|  |  |
| **Q3b** Before today were you aware that carabid beetles eat crop weed seeds such as dandelion, shepherds purse and chickweed? | Tickbox response, one could be selected of *Yes* or *No* |
| **Q4a** Do you believe that carabid beetles can make a significant contribution to Crop insect pest control? | Tickbox response, one could be selected of *Yes, No*, or *Not sure* |
| **Q4b** Do you believe that carabid beetles can make a significant contribution to Crop weed control? | Tickbox response, one could be selected of *Yes, No*, or *Not sure* |
| ***Section 2 The farm environment and conservation*** | |
| Statement on farm measures: *“There are many measures that may help to increase the overall abundance and number of different predatory species of carabid beetles.*  *Some of these involve including natural habitat in proximity to crop areas so that carabids have resources over time; some encourage their increased movement into the crop area; and some reduce the mortality associated with farm operations.”* | |
| **Q5** Have you implemented the following farm management? (AES= agri-environment schemes) | The response was in the form of a table with rows associated with the FMPs listed in Table 2 and the columns associated with the responses *(i)*  *In the past, through AES, (ii) In the past, voluntarily (iii) Currently, through AES, (iv) Currently, voluntarily (v) No/Not applicable.* Multiple columns could be selected for each FMP. |
| **Q6** Do you carry out any of the above [FMPs] particularly with the aim of increasing the abundance of carabid beetles and their associated natural-enemy pest control? If so could you indicate which and provide some details please. | *Yes* or *No* with Qualitative response facilitated by a text entry box. |
| **Q7** Which, if any, of the above options would you consider carrying out, or increasing the amount you do, in order to boost the abundance of carabid beetles and their associated natural-enemy pest control? | Qualitative response facilitated by a text entry box |
| **Q8** Is there any reason you would be apprehensive about implementing any of the above options? | Qualitative response facilitated by a text entry box |
| **Q9a** How important in your opinion is the following FMP to improving the control of crop pests by natural-enemies such as carabids? | The response was in the form of a table with rows associated with the FMPs listed in Table 2 and the columns associated with the responses *(i) Extremely important; (ii) Very important (iii) Moderately important-*  *Slightly important (iv) Not at all important (v) Not sure* |
|  |  |
| **Q9b** How difficult would you rate the following farm management, in terms of implementing it on your farm (in terms of cost, labour, knowledge, equipment, and time)? | The response was in the form of a table with rows associated with the FMPs listed in Table 2 and the columns associated with the responses *(i) Extremely difficult; (ii) Moderately difficult; (iii) Slightly difficult (iv)*  *Not at all difficult; (v) Not sure (vi) Impossible due to soil or landscape constraints (vii) Impossible due to legal or tenancy constraints.* |
| ***Section 3 Farmer attributes*** | |
| Information statement: *“To put your answers in context we would like to know about your farm enterprise. All answers are confidential and you will not be identifiable by your response.”* | |
| **Q10** What is your farm type? Please tick the box that most accurately describes your farming enterprise*.* | Tickbox response, one could be selected of 10 options, from Defra categories (Defra 2020a): *(i) Dairy; (ii) LFA/upland Grazing Livestock; (iii) Lowland Grazing Livestock; (iv) Cereals; (v) General cropping; (vi) Pigs; (vii) Poultry; (viii) Mixed; (ix) Horticulture; (x) Not applicable*  Classified for analysis as: Cereals; Livestock; General cropping; and Mixed |
| **Q11** What is the size of your farm? | Tickbox response, one could be selected of *(i) Under 20 hectares; (ii) 21 to 50 hectares; (iii) 51- 100 hectares; (iv) 101 - 500 hectares; (v) Over 500 hectares; (vi) Not applicable*  Classified for analysis as: Under 50 ha; 50-100ha; 100-500ha; and Over 500ha |
| **Q12** What are the sources of your farming experience and knowledge? Please tick all that apply  (multiple boxes can be checked) | Tickbox response, one could be selected of *(i) Farming background;*  *Farm work from childhood/ leaving school; (ii) College course/further education (agricultural); (iii) University level education (agricultural); (iv)*  *Agricultural industry qualification- e.g. BASIS*  Classified for analysis as: Non-formal education; Formal education; and Industry qualification |
| **Q13** Do you receive advice on farm management from any of the following? Please tick all that apply  *(*multiple boxes can be checked) | Tickbox response, one could be selected of *(i) Agricultural groups/bodies; (ii) Conservation organisations; (iii) Governmental organisations; (iv)Agronomists /professional advisors; (v) Industry representatives; (vi) Farm events/ training; (vii) Farmer networks/farming colleagues*  Classified for analysis as: top-down advice *(i)-(v)*, and participatory advice *(vi)* and *(vii)* |
